# Survival, causes of death, and cardiovascular events in patients with Marfan syndrome

**DOI:** 10.1002/mgg3.489

**Published:** 2018-11-04

**Authors:** Thy Thy Vanem, Odd Ragnar Geiran, Kirsten Krohg‐Sørensen, Cecilie Røe, Benedicte Paus, Svend Rand‐Hendriksen

**Affiliations:** ^1^ Institute of Clinical Medicine, Faculty of Medicine University of Oslo Oslo Norway; ^2^ Department of Cardiothoracic Surgery Oslo University Hospital Oslo Norway; ^3^ Department of Physical Medicine and Rehabilitation Oslo University Hospital Oslo Norway; ^4^ Department of Medical Genetics Oslo University Hospital Oslo Norway; ^5^ TRS, National Resource Centre for Rare Disorders Sunnaas Rehabilitation Hospital Nesoddtangen Norway

**Keywords:** aortic surgery, cardiovascular events, causes of death, Marfan syndrome, survival

## Abstract

**Background:**

To explore survival, causes of death, and the prevalence of cardiovascular events in a Norwegian Marfan syndrome (MFS) cohort. MFS is a heritable connective tissue disorder associated with reduced life expectancy–primarily due to aortic pathology.

**Methods:**

A follow‐up study of 84 MFS adults, initially investigated in 2003–2004. In 2014–2015, 16 were deceased, 47 of 68 survivors consented to new clinical investigations. Analyses of events were performed for 47 survivors and 16 deceased at follow‐up. Standardized mortality ratios (SMR), using the mortality rate of the Norwegian population as reference, were calculated for all 84 and calculated for men and women separately. Causes of death and information on cardiovascular events were retrieved from death certificates and medical records.

**Results:**

Standardized mortality ratios (95% confidence interval): for the whole cohort: 5.24 (3.00–8.51); for men: 8.20 (3.54–16.16); for women: 3.85 (1.66–7.58). Cardiovascular causes were found in 11 of 16 deceased, eight of these related to aortic pathology. Cancer was the cause of death in three patients. At follow‐up, 51% had new cardiovascular events; 59% had undergone aortic surgery. Men experienced aortic events at younger age than women. 32% of the survivors were not followed‐up as recommended.

**Conclusion:**

Life expectancy is reduced in this MFS cohort compared to the Norwegian population. Cardiovascular complications develop throughout life, particularly aortic pathology, the major cause of death in MFS. Death and aortic pathology seem to occur earlier in men. There is a need to improve follow‐up according to guidelines.

## 1 INTRODUCTION

Marfan syndrome (MFS), an autosomal dominant disorder of connective tissue caused by mutations in the fibrillin‐1 gene, *FBN1*, (OMIM *134797) is a potentially life‐threatening syndrome. Several reports indicate that lifespan is shortened (Murdoch, Walker, Halpern, Kuzma, & McKusick, 1972; Silverman et al., 1995) primarily due to increased risk of aortic pathology, such as aortic dilatation or dissection. Other conditions, such as valvular heart disease and myocardial dysfunction with arrhythmias are also known as causes of premature death in MFS (Yetman, Bornemeier, & McCrindle, 2003). Some studies indicate that men experience aortic diseases at younger age than women with MFS (Detaint et al., 2010; Groth et al., 2017; Rand‐Hendriksen et al., 2009).

Better diagnostics and medical and surgical treatment have increased life expectancy, as shown in several papers (Fuchs, 1997; Gray et al., 1998; Silverman et al., 1995). Different studies have shown different clinical history depending on the selection of MFS patients (Jondeau et al., 2012; Puluca, Burri, Cleuziou, Krane, & Lange, 2018). In patients with MFS without previous aortic surgery or dissections, follow‐up with strict surveillance and prophylactic measures, have provided excellent survival (Jondeau et al., 2012). The diagnostic criteria for MFS have been revised several times (Beighton et al., 1988; De Paepe, Devereux, Dietz, Hennekam, & Pyeritz, 1996; Loeys et al., 2010; Pyeritz & McKusick, 1979). The findings of presumed disease‐causing variants in a number of genes other than *FBN1* in persons formerly diagnosed with MFS, have given rise to new diagnoses of heritable connective tissue disorders (HCTD) with overlapping symptoms and clinical findings. The natural history and the influence of medical interventions on MFS are not fully described and there is lack of knowledge about age‐dependent penetrance of cardiovascular complications in MFS. Follow‐up studies of MFS cohorts describing the previous natural history or the current clinical history are missing.

As life expectancy increases, age‐dependent diseases in the general population will affect MFS patients, and may change the causes of death in the MFS population accordingly (Hasan, Poloniecki, & Child, 2016). Although current treatment might enhance survival, our main hypothesis is that life expectancy in an unselected MFS population is still significantly reduced compared to the general population, for a large part due to aortic pathology, but also due to other cardiovascular complications. Our second hypothesis is that aortic diseases are more frequent and still occurs at younger age in men than in women with MFS. The overall aim of this study is to assess survival, the causes of death and the prevalence of cardiovascular events in a Norwegian cohort of MFS patients, diagnosed according to the Ghent nosology from 1996 (Ghent‐1) (De Paepe et al., 1996), re‐examined after 10–12 years. We also wanted to evaluate whether or not the clinical follow‐up was in accordance to current guidelines (Erbel et al., 2014) in the follow‐up period, since Norway does not have follow‐up in large volume centers with experience in HCTD.

## 2 MATERIALS AND METHODS

### 2.1 Patients

**Figure 1:**
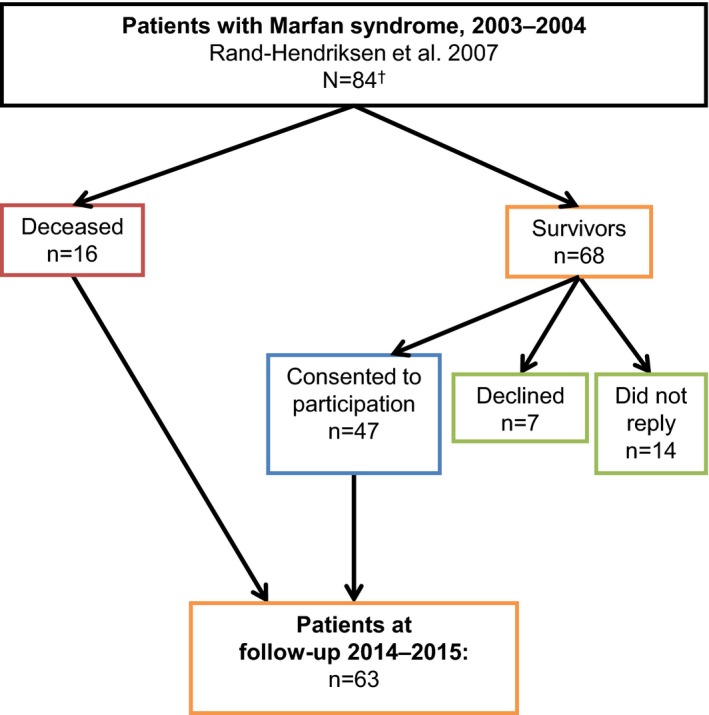
A flow chart of the study population. ^†^Due to new knowledge about disease‐causing genes, the MFS cohort from 2003–2004 has been reclassified from 87 MFS patients to 84 patients at follow‐up

The study is based on a cohort originated from a cross sectional study of 105 adults (≥18 years) with presumed MFS in 2003–2004 (Rand‐Hendriksen, 2010). The participants were recruited through the TRS, National Resource Centre for Rare Disorders, the Journal of the National Association for MFS and through the Department of Cardiothoracic Surgery at the University Hospital in Oslo by 1 January 2003. All the participants were assessed for all organ systems described in Ghent‐1. After the first investigations, 87 patients fulfilled the diagnostic criteria for MFS according to Ghent‐1 and initially 73 patients had a presumed pathogenic mutation in *FBN1* (Rand‐Hendriksen et al., 2007; Tjeldhorn et al., 2015). In 2013, high throughput sequencing analysis of a panel of 44 HCTD genes became available and clinical testing was performed in *FBN1* mutation negative patients. The GenBank reference sequence number was NM_000138.4. If a presumed disease‐causing variant was identified in a gene causing one of the types of Loeys‐Dietz syndrome (LDS), the patient was not considered to have MFS, irrespective of the fulfilment of the clinical criteria (Loeys et al., 2010). Thus after new genetic analysis, 84 of 87 MFS patients from the original cohort were diagnosed with MFS. All survivors and deceased in the cohort were identified from the National Registry (The Norwegian Tax Administration, 2018). In 2014, the survivors of the 84 MFS patients were invited to re‐investigations (Figure 1). Medical records, death certificates, and autopsy reports, where these had been performed, were collected. December 2015 was the closing date for the clinical follow‐up investigations. All the participating survivors were assessed for all organ systems described in Ghent‐1. In addition, at the second investigations the participants were interviewed about the place, frequency, and method of follow‐up during the 10–12‐year period.

### 2.2 Registration of deaths and other events

All deaths in Norway (about 40,700/year) are registered in the Cause of Death Registry at the Norwegian Institute of Public Health, based on the death certificate, issued by a physician, including the time, place, and assumed cause of death. Hospital records are continually updated with the date of death. Hence, for the follow‐up the deceased were identified through the hospital records and the National Registry until 31 December 2015. Death certificates and hospital records were obtained for all the deceased, and all accessible information about the health and treatment during the follow‐up period was assessed. Three of the authors reassessed together the causes of death, based on all the collected information. The cause of death was dichotomized as: “cardiovascular” or “noncardiovascular”. Due to a small cohort no risk adjustment for cardiovascular risk factors was performed.

All 84 MFS patients, according to Ghent‐1, were included in the analyses of survival and mortality. The Norwegian population of 5.28 million (August 2017) was used as a control group to compare mortality between the groups. Data on mortality rate in the Norwegian population was obtained from Statistics Norway.

The cause of death in the deceased and the prevalence of cardiovascular events for all the deceased and the survivors who participated in the follow‐up investigations were registered.

In order to compare with previous studies, we have defined two sets of events in this study: “aortic events” and “all cardiovascular events”, the latter also including aortic events. “Aortic events” were defined as: a new aortic dissection (Stanford type A or B), prophylactic and acute aortic surgery (in any parts of aorta). “Aortic event‐free survival” was defined as survival without occurrence of aortic events. By review of the hospital records for each patient and data from inclusion and follow‐up, the age of first occurrence of aortic events were collected. “All cardiovascular events” were defined as: a new aortic dissection (Stanford type A or B), prophylactic and acute aortic surgery (in any parts of aorta), mitral valve prolapse (with or without repair), arrhythmia requiring treatment, bacterial endocarditis and stroke (neurological deficit beyond 24 hr).

Other events such as other vascular pathology or pathology related to other organs, for example, the ocular system, often affected in MFS, were not included in these analyses.

### 2.3 Statistical analysis

*Survival* was calculated based on 84 MFS patients. Standardized mortality ratios (SMR) were calculated for all 84 and for men and women separately. SMR estimates exceeding 1.0 represent higher mortality rates in comparison to the general Norwegian population. The number of person‐years at risk for the MFS patients in age group intervals of 5 years was calculated and used to estimate the expected number of deaths in the general Norwegian population using Statistics Norway's age‐specific death rates for males and females (18). SMR is then the ratio between the observed numbers of deaths in the cohort of MFS patients and the expected numbers of deaths in a cohort with equal age and gender distribution from the general Norwegian population.

Aortic event‐free survival was calculated based on the living and deceased MFS patients included in the follow‐up study. We did not have knowledge about those who did not participate in the follow‐up study and could therefore not calculate for all the 84 MFS patients. Aortic event‐free survival was defined as the interval between the date of birth and the first registration of an aortic event in the medical records, since MFS is a congenital disorder and thus the risk of aortic events is assumed to start at birth.

The Kaplan‐Meier method was used to estimate the cumulative probabilities of survival and of aortic event‐free survival. The results are expressed with 95% confidence interval (CI). The log‐rank test was performed and *p*‐values of <0.05 were considered statistically significant.

The prevalence of all cardiovascular events is expressed as frequencies and percentages.

IBM Corp. Released 2017. IBM SPSS Statistics for Windows, Version 25.0. Armonk, NY: IBM Corp. was used for all statistical analyses, except for estimation of SMR conducted with StataCorp. 2015. *Stata 14 Base Reference Manual*. College Station, TX: Stata Press, using the istdize command.

The study was approved by the Regional Committees for Medical and Health Research Ethics, South East, Norway, registration number 2013/2109. The approval included follow‐up of the deceased and the consenting surviving patients.

## 3 RESULTS

### 3.1 Study population and overall results

**Figure 2:**
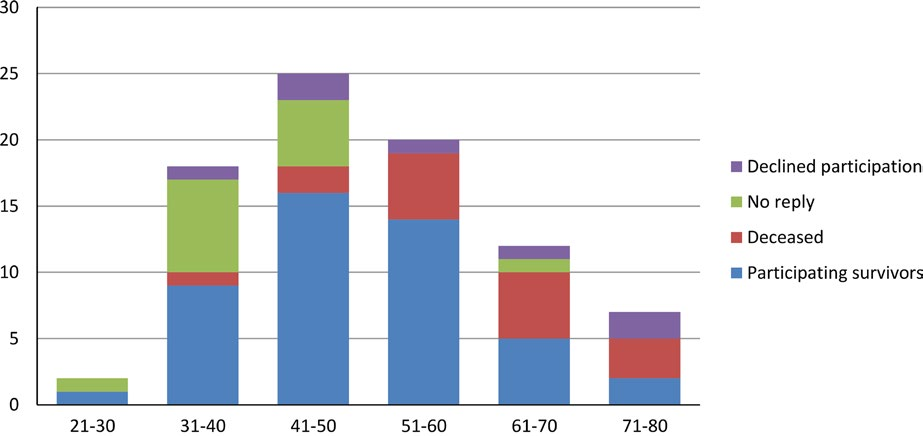
Age distribution of 84 MFS patients at 10–12‐year follow‐up: the *x*‐axis showing age groups of 10‐year intervals and the *y*‐axis showing the number of patients

Of 84 MFS patients, 16 were deceased (eight men and eight women) by 31 December 2015. Of 68 MFS survivors, 47 participated in the follow‐up investigations, 21 survivors did not reply or declined participation (Figure 1). The nonrepliers were seven males and seven females, age ranged from 29–64 years. Most patients who did not respond to the invitation for the follow‐up investigations were in the age group of 31–50 years. Those who declined, two males and five females, age ranged from 37–74 years, were evenly distributed in the group from 31–80 years (Figure 2). Thus, 63 MFS patients, 47 survivors and 16 deceased, were included in the analyses of aortic events and all cardiovascular events (For specific *FBN1* mutations, see supplementary table). For the deceased, median survival from inclusion in January 2003 were 9 years (range 3.5–12.5 years). For all 47 survivors, median follow‐up from inclusion in January 2003 until closing date of investigations in December 2015 were 11.5 years (range 11–12.5 years).

### 3.2 Survival and aortic event‐free survival

**Figure 3:**
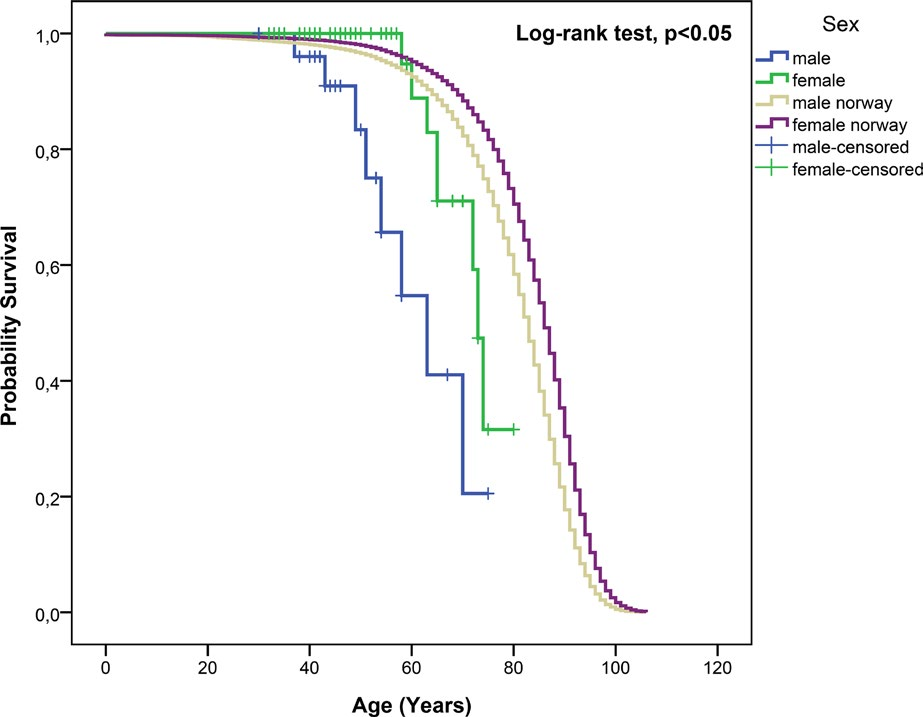
Cumulative probability of survival in 84 MFS patients compared to the general Norwegian population. Median estimate male: 63 years (95% CI: 51.3–74.7). Median estimate female: 73 years (95% CI: 70.8–75.2)

Standardized mortality ratios (95% CI) was 8.20 (3.54–16.16) for men and 3.85 (1.66–7.58) for women. This means that the MFS men have about eight times higher, and the MFS women have almost four times higher mortality compared with the general Norwegian population. For the whole MFS cohort SMR was 5.24 (3.00–8.51). The median cumulative probability of survival (the age at which 50% of the patients are predicted to still be alive in this MFS cohort; 95% CI) was 63 years (51.3–74.7) for men and 73 years (70.8–75.2) for women, which is significantly reduced compared to the general Norwegian population (Figure 3).

**Figure 4:**
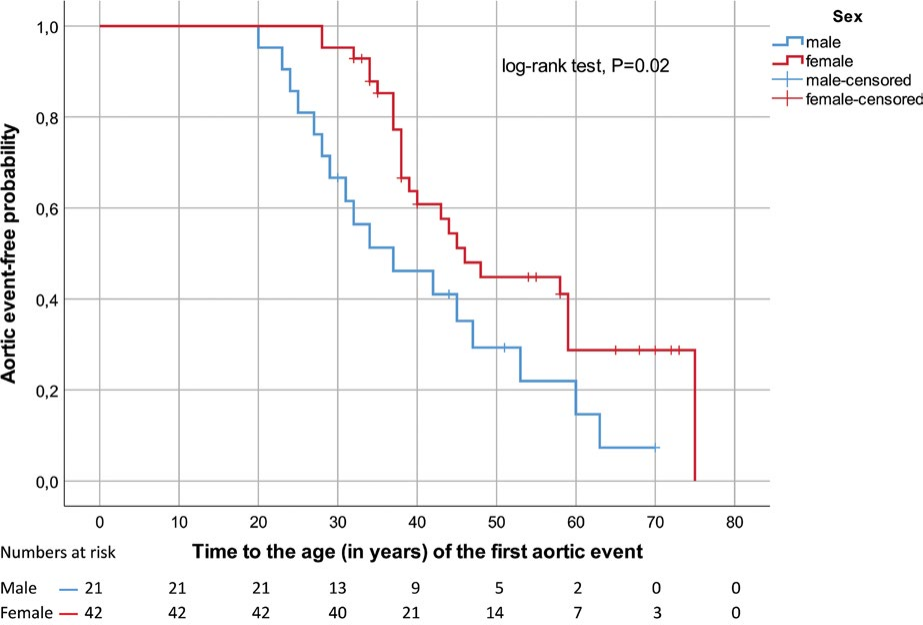
Aortic event‐free survival, 63 MFS patients

**Figure 5:**
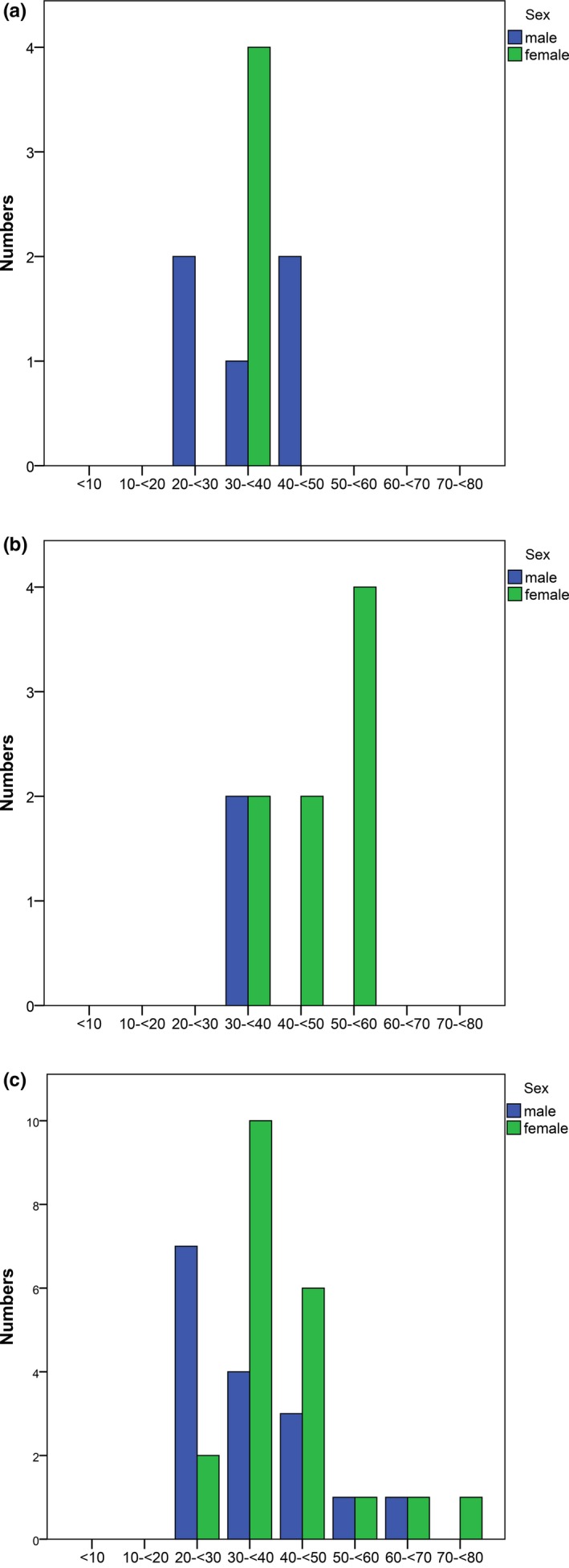
(a) Age at first occurrence of Stanford type A dissection, *N* = 63. (b) Age at first occurrence of Stanford type B dissection, *N* = 63. (c) Age at first time aortic surgery, *N* = 63

The median cumulative probability of aortic event‐free survival (when 50% are still alive and free of an aortic event; 95% CI) was for men 37 years (22.8–51.2) and for women 46 years (39.5–52.5; Figure 4). Figure 5a–c, illustrates at which age of patients in the cohort of 63 MFS, survivors, and deceased, were first diagnosed with aortic dissection and the age of the first aortic surgery. The figures show a left shift for men regarding age. The number of type B dissections is about the same as for type A dissections. All patients diagnosed with type B dissections were initially treated medically, but one of these needed surgical treatment after the initial medical treatment due to rupture of the descending aorta. Additional four patients underwent surgery in the descending aorta several years after the event of B dissection due to progression. More men than women in the age group 20–30 years, underwent aortic surgery for the first time. The differences in survival between the genders were statistically significant with *p*‐value <0.05. For aortic event‐free survival, the gender differences were statistically significant with *p*‐value = 0.02. Of the 16 deceased, only two did not experience any cardiovascular events before death.

### 3.3 Causes of death

A death certificate was available for all 16. Table 1 reports the causes of death based on the information from the death certificates, the medical records and reassessments by three of the authors and dichotomized as “cardiovascular” or “noncardiovascular”. Three out of 16 (19%) deceased were diagnosed with mitral valve prolapse and 14 of 16 (88%) were diagnosed with dilatation or dissection in the aortic root/ascending aorta during life. One of the two patients who was not diagnosed with aortic dilatation or dissection (Table 1, no 15 and 16) had a child with aortic dilatation. The other had a bicuspid aortic valve.

**Table 1:** Characteristics of the 16 deceased

No	Age		Aorta					Causes of death	
	At FI	At death	Before FI		After FI			Cardiovascular	Non‐cardiovascular
			Dissection^a^	Surgery	Dissection^a^	Surgery	New pathology^b^		
1	33	37	No	No	No	Yes	Yes	Postoperative aortic dissection^a^	
2	40	43	No	Yes	Type A	Yes	Yes	Postoperative aortic dissection^a^	
3	54	65	No	No	Type B	No	Yes	Stroke probably due to aortic dissection^a^	
4	53	63	No	No	No	No	No	Heart failure (AI+MR)	
5	39	51	No	No	No	No	Yes	Heart failure (AI +coronary heart disease )	
6	65	74	No	Yes	No	No	Yes	End‐stage heart failure due to AI	
7	53	60	No	Yes	No	No	No	Multiple organ failure/brain injury after heart transplant	
8	47	54	No	Yes	No	No	No	Complications due to aortic dilatation? Arrhythmia?	
9	45	58	No	No	No	No	Yes	MI/arrhythmia/aortic pathology?	
10	58	63	Type B	No	No	No	No	Cardiac arrest	
12	48	58	Type A	Yes, twice	No	No	No	Septicemia and DIC (possible endocarditis)	
11	40	49	No	Yes	No	No	No		Colon cancer with metastases
14	68	73	No	No	No	No	No		Colon cancer with metastases
13	61	72	No	No	No	No	Yes		Septicemia
15	58	70	No	No	No	No	No		Septicemia
16	60	65	No	No	No	No	No		Non‐Hodgkin lymphoma

Notes

AI: aortic insufficiency; DIC: disseminated intravascular coagulation; FI: The first investigations; MI: myocardial infarction; MR: mitral regurgitation.

a Stanford type A/B.

b New/progression of aortic pathology.

Eleven of the 16 deaths were due to cardiovascular causes, of which eight were related to aortic complications, including valve regurgitation due to dilatation. Six of 11 patients, who died of cardiovascular causes, had undergone aortic surgery, one was previously diagnosed with Stanford type A dissection and one was diagnosed with Stanford type B dissection at inclusion to the study in 2003–2004. The medical records describe nine cancer‐related disorders in seven of 16 patients. Three of the deaths were caused by cancer.

### 3.4 The prevalence of all cardiovascular events

At the first investigation, 28 of 84 (33%) had undergone aortic surgery. Before inclusion in 2003, 17 patients underwent prophylactic aortic surgery only, while three patients underwent acute aortic surgery only. Five patients underwent both prophylactic and acute aortic surgery before inclusion. During the follow‐up period, 22 patients underwent prophylactic surgery and two patients experienced both prophylactic and acute surgery. At follow‐up, a total of 37 of 63 (59%) patients had undergone aortic surgery (Table 2). The oldest patient who underwent repair of the ascending aorta for the first time was 75 years old. Seven of the deceased underwent aortic surgery. Another seven of the deceased were diagnosed with aortic dilatation, but did not have any aortic surgery during their lifetime, two of them having a Stanford type B dissection. One of the deceased who underwent aortic surgery, a mechanical valved conduit, before inclusion, experienced a Stanford type A dissection 11 years after the first aortic surgery. Although it was indicated, surgery was not possible in one patient due to severe scoliosis and chest deformity. In another patient, surgery was not performed due to heart failure. During follow‐up 32 of 63 (51%) experienced new cardiovascular events.

**Table 2:** Characteristics of the Norwegian MFS cohort from the first investigations in 2003–2004 to follow‐up in 2014–2015

	2003–2004 *n* (%)	2014–2015 *n* (%)
*FBN1* mutation	55 (87)	58 (92)
Patients on β‐blockers and/or other antihypertensive agents	32 (51)	46 (73)
Patients with aortic surgery *before* inclusion in 2003	24 (38)	
Patients with new cardiovascular events		32 (51)
Patients with aortic surgery during follow‐up		24 (38)
Total patients who have undergone aortic surgery		37 (59)

Note

*N* = 63 (47 survivors and 16 deceased), 42 (67%) women.

Table 3 shows the characteristics of the MFS survivors. Twenty‐six out of 47 (55%) had experienced new cardiovascular events with a total of 56 cardiovascular events, 40 of which were aortic events. One of the five aortic dissections occurred postpartum. Only 34 of 47 survivors (72%) were treated with β‐blockers and/or angiotensin II receptor blockers.

**Table 3:** Characteristics of the 47 MFS survivors at follow‐up, 2014–2015

	*n* (%)
Women	34 (72)
*FBN1* mutation	45 (96)
Patients with aortic surgery *before* inclusion in 2003	18 (38)
Total patients who have undergone aortic surgery	30 (64)
β‐blockers and/or other antihypertensive agents	35 (74)
Inadequate follow‐up during the follow‐up period	15 (32)
Patients with new cardiovascular events:	26 (55)
Patients with aortic surgery during the follow‐up period	22 (47)
Patients with a new Stanford type A or B dissection	5 (11)
Mitral valve prolapse with/without surgery	2 (4)
Arrhythmia	6 (13)
Bacterial endocarditis	1 (2)
Stroke	4 (9)

### 3.5 Follow‐up of the survivors during the 10–12‐year period

After the first investigations, all patients, their local general physicians, cardiologists and ophthalmologists received a complete medical report with information about the need of systematic follow‐up. The recommended frequency and method of follow‐up was outlined for all. In spite of this, 15 of 47 (32%) were not followed‐up as recommended. One survivor had no follow‐up over the whole period. In the 32 (68%) patients who were adequately followed‐up, CT or MRI of the aorta and echocardiography were performed regularly, that is, twice a year, once a year or every second year, depending on the progression of their aortic disease. As a consequence of the second investigations, five of 47 (11%) were referred to evaluation for prophylactic surgery. These patients had not been referred during the follow‐up period in spite of aortic root diameter of ≥5 cm and family histories of severe aortic pathology.

## 4 DISCUSSION

In our cohort of adults with MFS, the main finding is that in spite of available medical and surgical interventions, life expectancy is significantly reduced in the whole MFS cohort compared to the general Norwegian population of 5.28 million, not adjusted for cardiovascular risk factors. This is primarily due to aortic complications. Compared to gender and age‐matched groups in the general Norwegian population, men in this cohort had about eight times higher risk of death, while women in this cohort had about four times higher risk. Men had a median age of survival of 63 years compared to 73 years in women in this MFS cohort. Compared to the study of Fuchs (1997 from 1997, on a Danish cohort where the information was provided from medical records and files and not on clinical investigations, the cumulative probability of survival for men in our study is higher (median 63 years) compared to 57 years in the Danish study. Our study shows that the cumulative probability of survival for women with MFS is also higher, 73 years in this Norwegian cohort, compared to 58 years as reported by Fuchs et al. The survival rate in our study might be too high compared to the general MFS population, since only living patients ≥18 years of age were included in 2003.

This study also supports previous findings that men seem to experience aortic events at younger age than women. Although men only accounts for one third of the cohort, more men in the younger age group experience aortic events compared to women in this study. There is a predominance of first time aortic events in men in the age group from 20 to 30 years. Previous studies have also shown that aortic events occur earlier in men than women (Detaint et al., 2010; Faivre et al., 2007; Groth et al., 2017; Rand‐Hendriksen et al., 2009). The reason for the gender differences of men undergoing aortic surgery at a younger age, parallel to a shortened life expectancy, is not known.

The study supports the hypothesis that adults with MFS have a progressive aortic disease throughout life. In our study, the oldest patient who underwent aortic surgery in the ascending aorta for the first time was 75 years old and patients with previous prophylactic aortic surgery have experienced consecutive aortic surgeries. This is consistent with the findings in the study of Detaint et al. (2010 and Puluca et al. (2018. It is a disturbing observation that only 70% of the patients in this study were treated with β‐blockers and/or angiotensin II receptor blockers. A significant number of aortic events occurred during the period between the first investigations and at follow‐up. According to current guidelines, therapy with β‐blockers is recommended in all adults who are diagnosed with MFS, regardless of the dimensions of the aortic root, that is, whether or not the root is dilated. At the first investigations about 40% of the MFS patients had undergone aortic surgery compared to almost 60% at follow‐up. In accordance to current guidelines (Erbel et al., 2014; Guidelines for the management of grown‐up congenital heart disease (new version (2010)), 2011) five of the survivors were referred to evaluation for prophylactic surgery as a consequence of the follow‐up.

This study shows that 32% of the survivors were not followed up and treated according to the written advice and current guidelines. Medical service for patients with MFS/HCTD is not centralized in Norway and people are living scattered in rural areas and patients are seen at local and regional hospitals. Some hospitals might not have health care professionals with sufficient knowledge of MFS/HCTD. This might partly explain the inadequate follow‐up some of the patients have experienced. In one study on MFS patients without previous aortic surgery or dissections, the long term survival was excellent and this observation should guide future organization of the MFS care (Jondeau et al., 2012). A centralized follow‐up of all MFS patients at a HCTD centre in close cooperation with the local hospital and general practitioner might improve follow‐up and treatment, since also specialized surgery might be needed in the long‐term care of the patients.

### 4.1 Strength and limitations

The strength of this study is that all patients have been examined for all features and organ systems that are described in the diagnostic criteria, assuring high validity for MFS. We have a fairly homogenous diagnostic workup, since genetic sequencing was also performed for all patients. In previous studies, the study population may have included individuals with several HCTD, not only MFS. A weakness of the study is that the representativeness is uncertain, in particular because of the female preponderance. Due to a small study population we have only included age and sex when comparing aortic events between females and males and not adjusted for cardiovascular risk factors, which might influence the results. The minimal age of inclusion in 2003 was 18 years or above, thus patients with a severe phenotype of MFS and premature death might have been missed. Persons with milder phenotypes and thus not yet diagnosed may also be missed and not invited to this study. This group would have increased the life expectancy in our MFS cohort.

## 5 CONCLUSION

Life expectancy is reduced in this Norwegian MFS cohort compared to the Norwegian population, although life expectancy for women with MFS is higher compared to a study of another Scandinavian cohort two decades ago (Fuchs, 1997). Cardiovascular complications, in particular aortic disease, seem to still be the main cause of premature death in patients with MFS. Hence, it is crucial that persons with MFS are diagnosed early and are followed up and treated regularly throughout life according to the present guidelines. As lifespan is expected to increase, other age‐dependent conditions may increasingly contribute to the causes of death. More than 90% of the MFS cohort had developed aortic pathology at 10–12‐year follow‐up. Men seem to experience aortic events at younger age than women. Fifty‐one percent experienced a new cardiovascular event at follow‐up. Cardiovascular pathology will probably progress throughout life.

## CONFLICT OF INTEREST

The authors declare no conflict of interest.
