# Supplementary material for: Survival, causes of death, and cardiovascular events in patients with Marfan syndrome
Source: Mol Genet Genomic Med. 2018 Nov 4;6(6):1114–23. doi: 10.1002/mgg3.489 (PMC6305663; doi:10.1002/mgg3.489)
Supplement: Supplementary file 1 [file MGG3-6-1114-s001.pdf]

Supplementary table

Mutations in *FBN1* (*134797) in 58 MFS subjects

Nucleotide change	Interpretation
c.266G>A	p.Cys89Tyr
c.618delA	p.Lys207fs
c.629G>A	p.Cys210Tyr
c.1090C>T	p.Arg364X
c.1585C>T	p.Arg529X
c.2170A>G	p.Ile724Val
c.2305T>A	p.Cys769Ser
c.2308delG	p.Val770frX
c.2447G>T	p.Cys816Phe
c.2848T>C	p.Cys950Arg
c.3083-2A>G	Splice site mutation
c.3338A>G	p.Asp1113Gly
c.4087G>A	p.Asp1363Asn
c.4211-1G>A	Splice site mutation
c.4269_4270delAC	p.Ala1423fs
c.4348T>G	p.Cys1450Gly
c.4588C>T	p.Arg1530Cys
c.4620_4621dupGACCT	p.Arg1541fs
c.4925A>G	p.Asp1642Gly
c.5296G>C	p.Asp1766His
c.5559del(T)	p.Cys1853fs
c.5866T>C	p.Cys1956Arg
c.6388G>A	p.Glu2130Lys
c.6508T>A	p.Cys2170Ser
c.7094G>A	p.Cys2365Tyr
c.7168T>A	p.Cys2390Gly
c.7421G>A	p.Tyr2474Cys
c.7816_7817delGT	p.Val2606fs
c.7886A>G	p.Tyr2629Cys
c.8422C>T	p.Gln2808X
c.247+1G>A	Splice site mutation
c.4816+3delAATTGT	Splice site mutation
c.4942+2T>C	Splice site mutation
c.5788+5G>A	Splice site mutation
Del exon 1-6	Large deletion
Del exon 3	Large deletion
Del exon 63-65	Large deletion
